# SOST Deficiency Aggravates Osteoarthritis in Mice by Promoting Sclerosis of Subchondral Bone

**DOI:** 10.1155/2019/7623562

**Published:** 2019-11-11

**Authors:** Jingyu Li, Junjie Xue, Yan Jing, Manyi Wang, Rui Shu, Hui Xu, Chaoran Xue, Jie Feng, Peiqi Wang, Ding Bai

**Affiliations:** ^1^Department of Stomatology, Beijing Tiantan Hospital, Capital Medical University, Beijing 100070, China; ^2^State Key Laboratory of Oral Diseases & National Clinical Research Center for Oral Diseases & Department of Orthodontics and Pediatric Dentistry, West China Hospital of Stomatology, Sichuan University, Chengdu 610041, China; ^3^Department of Orthodontics, Beijing Stomatological Hospital, Capital Medical University, Beijing 100050, China; ^4^Department of Orthodontics, Texas A&M College of Dentistry, Dallas 75246, USA

## Abstract

As the initial part in the development of osteoarthritis (OA), subchondral bone sclerosis has been considered to be initiated by excess mechanical loading and proven to be correlated to other pathological changes. Sclerostin, which is an essential mechanical stress response protein, is encoded by the SOST gene. It is expressed in osteocytes and mature chondrocytes and has been proven to be closely correlated to OA. However, the relationship and mechanism between the SOST gene and the development of OA remain unclear. The aim of the present study was to investigate the role of the SOST gene in OA pathogenesis in the subchondral bone. A knee anterior cruciate ligament transection (ACLT) mouse osteoarthritis (OA) model on SOST-knockout (SOST KO) and wild-type (WT) mice was established. The pathogenic and phenotypic changes in the subchondral bone were investigated by histology, micro-CT, immunohistochemistry, TRAP staining, Masson staining, and Toluidine blue staining. It was found that sclerostin expression decreased in both the calcified cartilage and mineralized subchondral structures during the development of OA. Joint instability induced a severe cartilage degradation phenotype, with higher OARSI scores in SOST KO mice, when compared to WT mice. SOST KO mice with OA exhibited a higher BMD and BV/TV ratio, as well as a higher rate of bone remodeling and TRAP-positive cell number, when compared to the WT counterparts, but the difference was not significant between the sham-operation groups. It was concluded that loss of sclerostin aggravates knee OA in mice by promoting subchondral bone sclerosis and increasing catabolic activity of cartilage.

## 1. Introduction

Osteoarthritis (OA) is a degenerative joint disease, and the main pathological features are cartilage degradation, subchondral bone sclerosis, and osteophyte. The pathophysiological mechanism of the cartilage degradation of OA has been widely considered to be closely correlated to bone under mechanical loading [1]. However, as a whole joint disease, all cell types within the articular cartilage and its neighboring tissues are involved [2]. Abnormal subchondral bone remodeling and the interaction between cartilage and the underlying subchondral bone have been considered to be more important and significant in OA [3].

SOST/sclerostin is a canonical Wnt antagonist primarily synthesized by mature osteocytes and hypertrophic chondrocytes and functions as an osteogenesis inhibitor [4]. It has been considered as an important mediator of mechanical loading-induced new bone formation [4–8]. SOST gene mutation in human causes Van Buchem disease or sclerosteosis, which are both characterized as hyperostosis [9, 10]. Also, studies have shown that targeting this protein with a sclerostin-neutralizing monoclonal antibody is currently being developed as a new therapy for osteoporosis [5, 11].

SOST/sclerostin was previously implicated in OA pathogenesis [12, 13]. However, the precise effect of the SOST gene in OA is in need of further exploration. The dispute of this controversy mainly lies in the conflicting role of the SOST gene in bones and cartilage. The pathogenesis of OA is closely correlated to joint loading, and studies have shown that SOST is elevated in the cartilage but decreased in the subchondral bone in OA, suggesting opposing effects through the promotion of disease-associated subchondral bone sclerosis, while inhibiting the degradation of cartilage [12]. It has also been reported that sclerostin inhibits both Wnt canonical and noncanonical c-Jun N-terminal kinase (JNK) pathways, resulting in the maintenance of chondrocyte metabolism. As concluded by Chang et al. [14] the balance between the anabolic role of SOST in cartilage and the catabolic role of SOST in bones may be beneficially manipulated to promote favorable outcomes in posttraumatic OA (PTOA). Although the researches above showed similar results, the evidence still cannot fully explain the complexing role of SOST in development of OA.

In the present study, it was hypothesized that sclerostin plays a protective role in the development of OA through the negative control of subchondral bone osteogenesis and plays an anabolic role in cartilage, which is enhanced through the over loading of the joint at the early stage of the disease. A knee instability model was constructed to induce OA in wild-type (WT) and SOST gene knockout (SOST KO) mice and found a severer OA phenotype in SOST KO mice, in which bone formation in the subchondral unit increased only when stress was loaded, indicating the stress-dependent protective role of sclerosis on the early stage of OA.

## 2. Materials and Methods

### 2.1. Animal Models

Twenty 10-week-old male C57BL/6 mice (obtained from the Experimental Animal Center of Sichuan University, with a weight range of 20 ± 3 g) and 20 male complete SOST KO mice (kindly supplied by Professor Jian Q Feng from Baylor College of Dentistry, with a weight range of 20 ± 3 g) were selected for the present study.

The 20 WT mice were randomly divided into four groups, while the 20 SOST KO mice were randomly divided into two groups. The surgeries were performed under aseptic conditions. Anterior cruciate ligament transection (ACLT) was utilized to induce instability in the OA model on the right knees, as previously described [15]. The sham operation was performed on the left knees (single cutaneous incision and stitching) [16]. WT mice were sacrificed via excessive anesthesia at week 0 (*n* = 5), 2 (*n* = 5), 4 (*n* = 5), and 6 (*n* = 5) after surgery. SOST KO mice were sacrificed at week 0 (*n* = 10) and 4 (*n* = 10). The harvested mice knees were fixed, decalcified (for 20 days), dehydrated, and imbedded in paraffin. The continuous tissue sagittal sections were cut at a thickness of 4 *µ*m and used for all staining procedures.

Mice were maintained on a light-dark cycle (12 hours of light and 12 hours in the dark, with lights on at 8:00 AM) at a room temperature of 23 ± 1°C and provided with standard food and water *ad libitum*. All mice were cared for in accordance with the international standards for animal welfare, compliant with the guidelines of the Animal Care and Ethics Committee of West China School of Stomatology, Sichuan University (No. SKLODLL2013A172).

### 2.2. Histology

#### 2.2.1. Immunochemistry Staining

Immunochemistry staining was performed on the sagittal sections using anti-sclerostin antibody (1 : 200 diluted in PBS; R&D systems, MN, USA), anti-type-II collagen antibody (1 : 100 diluted in PBS; R&D systems, MN, USA), and anti-matrix metalloproteinase-13 (anti-MMP-13) antibody (1 : 200 dilution; Abcam). The staining was carried out according to manufacturer's instructions. The images of each staining were captured under a light microscope at the same setting. The percentage of sclerostin-positive cells over the total cells was measured by Image-Pro Plus (5.0, Media Cybernetics, Bethesda, MD).

#### 2.2.2. Tartrate-Resistant Acid Phosphatase (TRAP) Staining, Toluidine Blue, Hematoxylin and Eosin (H&E) Staining, and Masson Trichrome Staining

TRAP staining was used to measure the osteoclast genesis activity and was carried out using a leukocyte acid phosphatase kit (Sigma, USA), according to the instructions in the product datasheet. The TRAP-positive cells in the subchondral bone area were quantified as osteoclast numbers using the Image-Pro Plus software. Toluidine blue staining, H&E staining, and Masson trichrome staining were carried out according to the instructions of the product datasheet.

A histologic scoring system for murine OA, which utilized the toluidine blue staining of the Safranin O/fast green staining, known as the osteoarthritis research society international (OARSI) scoring, with a total severity score ranging from 0 to 12 [17], was applied to quantify the damage of the articular cartilage in both OA and sham knees. The scoring was performed by two blinded observers.

### 2.3. Microcomputed Tomography (CT) Scanning

**Figure 1:**
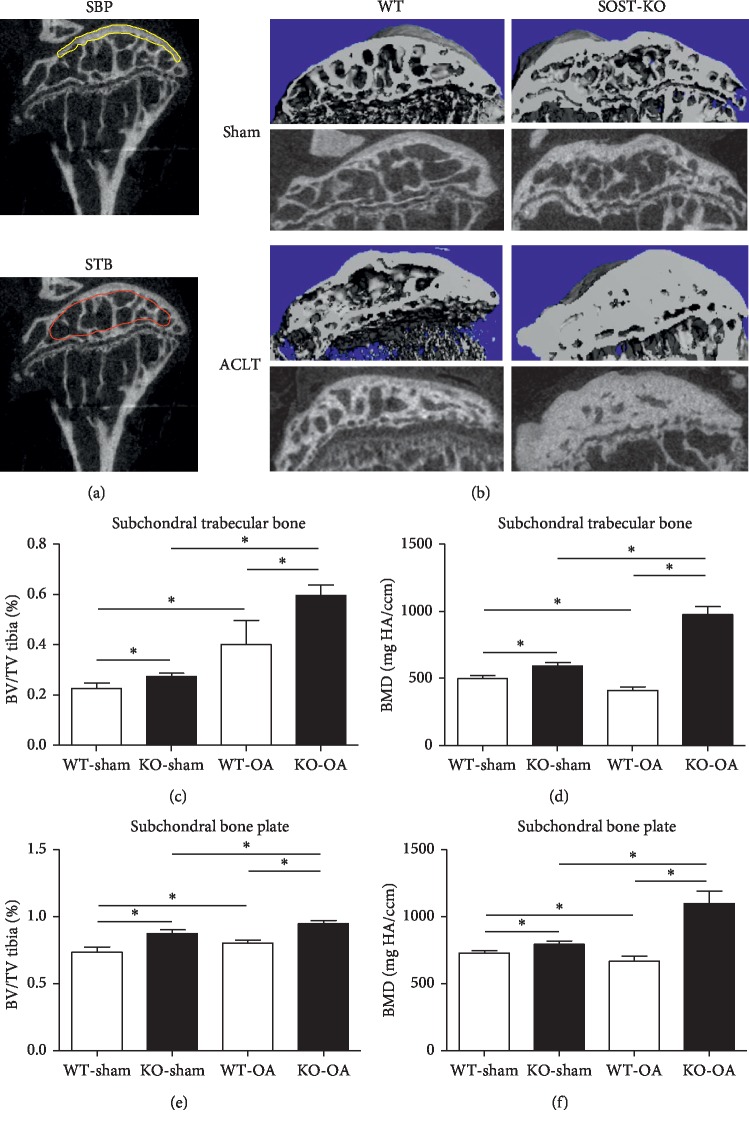
The BMD and BV/TV ratio in both the subchondral bone plate and trabecular bone in OA significantly increased in SOST KO mice, when compared to WT mice. (a) The area selected to analyze the BMD and BV/TV ratio of the SBP and STB (yellow line: subchondral bone plate; red line: subchondral trabecular bone). (b) The micro-CT 3D and 2D sagittal images of the OA and sham knees of SOST KO and WT mice show the changes of the subchondral bone plate and subchondral trabecular bone. (c–f) The BMD and BV/TV ratio of SOST KO mice significantly increased in both the SBP and STB after ACLT surgery (^*∗*^*P* < 0.05; the data were presented as mean ± SD).

The harvested samples were dissected free of soft tissues and fixed in 4% paraformaldehyde at 4°C for 24 hours. For the micro-CT analysis, the samples were imaged using a micro-CT scanner (Scanco, Viva40, Switzerland). The scanning range was the entire knee (including the tibia and femur, 5 mm of each), and the analyzed region was the subchondral bone plate and subchondral trabecular bone of the tibia plateau (Figure 1(a)). The energy setting for the scanning was 55 kV, the current was 145 *µ*A, and exposure time was 1,180 ms. Thirty consecutive slices of each sample were included, the region of interest was circled, and the bone mineral density (BMD) and bone volume (BV)/total volume (TV) value were analyzed using the built-in software.

### 2.4. Statistical Analysis

Data were expressed as mean ± standard deviation. The statistical analysis was performed using SPSS 17.0. All results were presented as the mean and standard deviation and assessed by independent Student's *t*-test or one-way ANOVA, when comparing data among the four groups. *P* < 0.05 was considered statistically significant.

## 3. Results

### 3.1. Sclerostin Was Found in Calcified Cartilage, Subchondral Bone Plate, and Subchondral Trabecular Bone

**Figure 2:**
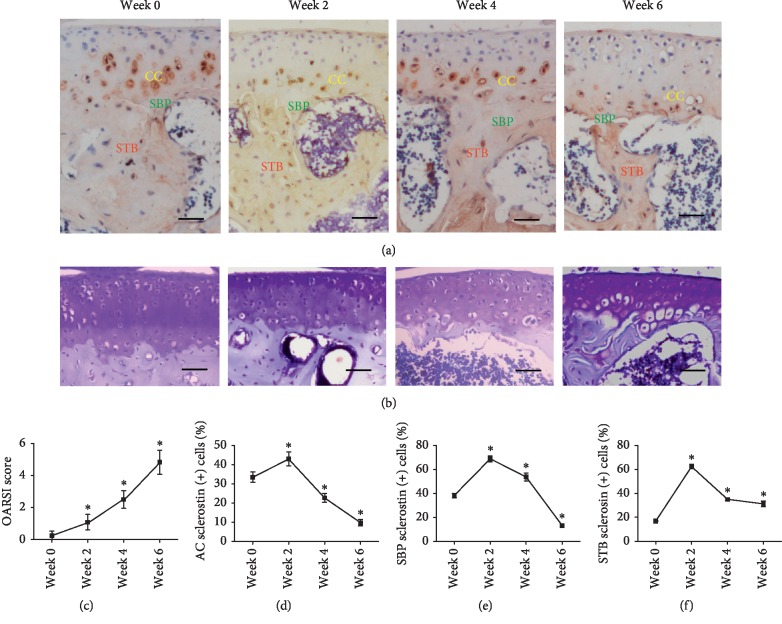
Expression trend of sclerostin in the CC, SBP, and STB of mice after ACLT. (a) The immunohistochemical staining of sclerostin in CC, SBP, and STB. CC: calcified cartilage; SBP: subchondral bone plate; STB: subchondral trabecular bone. (b) The toluidine blue staining revealed that OA in the tibial plateau was time dependently aggravated after ACLT in mice. Bar, 100 *µ*m. (c) OARSI scores of knee samples increased overtime after ACLT. (d–f) The percentage of sclerostin-positive cells in the CC, SBP, and STB. The expression of sclerostin in the CC, SBP, and STB exhibited the same trend, which increased from week zero to week two and decreased from week two to week six (^*∗*^*P* < 0.05*vs.* week zero; the data were presented as mean ± standard deviation (SD)).

Sclerostin expression was identified in mineralized tissue, including calcified cartilage, subchondral bone plate, and subchondral trabecular bone (Figure 2(a)), with a similar expression pattern during the 0–6 week time frame. The percentage of sclerostin-positive cells in the indicated zone sharply increased at 0–2 weeks after the onset of OA and decreased over time within 2–6 weeks in the calcified cartilage, subchondral bone plate, and subchondral trabecular bone (Figures 2(d)–2(f)). The expression patterns of sclerostin in osteocytes and chondrocytes during OA indicate the close correlation between sclerostin and the degradation of articular cartilage. The toluidine blue staining revealed that the degradation of the articular cartilage was aggravated in a 0–6 week time frame in OA (Figure 2(b)). The severity of OA was measured by OARSI scoring, and the scores increased over time according to OARSI scores (Figure 2(c)).

### 3.2. SOST KO Mice Developed a Severer OA Phenotype Shortly after ACLT

**Figure 3:**
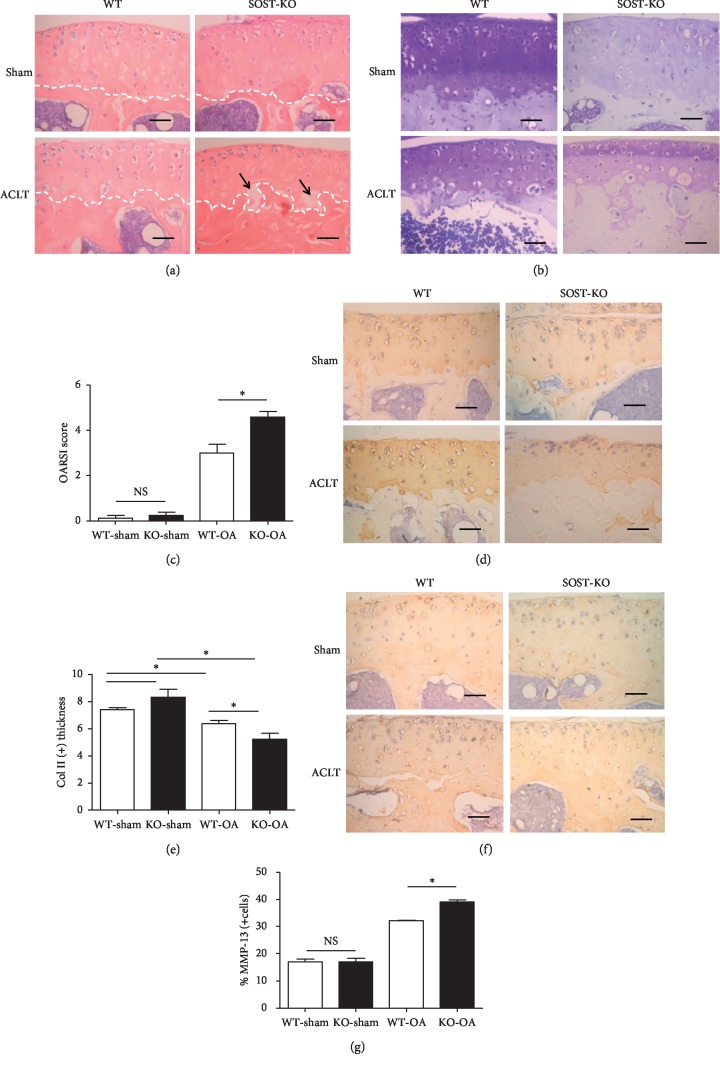
After ACLT, SOST KO mice presented with a severer OA phenotype, when compared to WT mice. (a) The H&E staining revealed different pathologic changes among SOST KO and WT mice. After ACLT, the contour of the tidemark (white dotted line) in OA knees of SOST KO mice became significantly distorted, when compared to that of WT mice. In addition, microcracks (black arrow) between the calcified and noncalcified structure occurred in OA knees of SOST KO mice, but this was not found in WT mice. Bar, 100 *µ*m. (b, c) OARSI scoring is applied by utilizing toluidine blue staining. No significant difference between the sham knees of SOST KO and WT mice was discovered. On the OA side, the score was significantly higher in SOST KO mice, when compared to WT mice. Bar, 100 *µ*m (*P* < 0.05; the data were presented as mean ± SD). (d, e) The immunohistochemical staining of type-II collagen presented with collagen loss of the OA knees. There was no significant difference between the sham knees of SOST KO and WT mice. On the OA side, the expression of type-II collagen in SOST KO mice was significantly lower than that in WT mice. Bar, 100 *µ*m (*P* < 0.05; the data were presented as mean ± SD). (f, g) The immunohistochemical of MMP-13 presents the catabolic activity in the cartage matrix of OA knees. There was no significant different between the sham knees of SOST KO and WT mice. On the OA side, MMP-13 was significantly higher in SOST KO mice than in WT mice. Bar, 100 *µ*m (^*∗*^*P* < 0.05; the data were presented as mean ± SD).

OARSI scoring, which is a histologic scoring system for murine OA that utilized toluidine blue staining, was applied to quantify the damage of the articular cartilage in the knee OA or sham control. No significant difference in cartilage loss was found between the SOST KO and WT mice with the sham surgery (Figure 3(c)). A severer cartilage degradation was found in SOST KO mice, with OARSI scores of 4.60 ± 0.55, when compared with the 2.98 ± 0.82 OARSI score in WT mice (*P* < 0.05; Figures 3(b) and 3(c)).

H&E staining was used to observe the histological characteristics of both the cartilage and surface of the subchondral bone plate. The contour of the tide mark, which separates the calcified cartilage from the noncalcified cartilage, was significantly distorted in SOST KO mice, when compared with WT mice, indicating that a more subchondral bone advanced into the articular cartilage in SOST KO mice (Figure 3(b)). In addition, significant cracks were observed between calcified and noncalcified tissues beside the tide mark in the OA knees of SOST KO mice, while no obvious cracks were observed in the OA knees of WT mice (Figure 3(a)).

Cartilage loss was detected by the immunochemical staining of type-II collagen. The expression of type-II collagen was lower in ACLT SOST KO mice, when compared to the ACLT WT mice (Figures 3(d) and 3(e)), suggesting that sclerostin may inhibit the shift towards a hypertrophic phenotype and aggravate the loss of cartilage collagen. The catabolic activity of the extracellular matrix in the cartilage was evaluated by the immunochemical staining of MMP-13, which functions as a catabolic enzyme [18, 19]. A significantly higher expression of MMP-13 was found in the cartilage of SOST KO mice, when compared to that in WT mice (*P* < 0.05, Figures 3(f) and 3(g)).

As indicated above, SOST deficiency resulted in a severer OA phenotype characterized by higher OARSI scores, the distortion of the tide mark, greater loss of cartilage collagen, and more disintegration of the extracellular matrix in mouse knees, suggesting that SOST/sclerostin may aggravate the development of OA by promoting the catabolic activity in cartilage.

### 3.3. SOST Deletion Increased Bone Formation in Both the Subchondral Bone Plate and Trabecular Bone in OA

The BMD and BV/TV of the subchondral bone plate and subchondral trabecular bone in both sham knees and OA knees of SOST KO mice and WT mice were separately evaluated in the present study. The selected micro-CT scanning area of the subchondral bone plate and subchondral trabecular bone is presented in Figures 1(a) and 1(b) (SBP: yellow line circled area, STB: red line circled area).

The loss of sclerostin resulted in higher BMD and a significantly increased BV/TV ratio in both the subchondral bone plate and trabecular bone (*P* < 0.05; Figures 1(c) and 1(d)), when compared to the WT counterparts, and in the sham-operated or ACLT groups.

### 3.4. SOST KO Mice Exhibited Increased Osteogenic Activity and Decreased Osteoclastic Activity in OA

**Figure 4:**
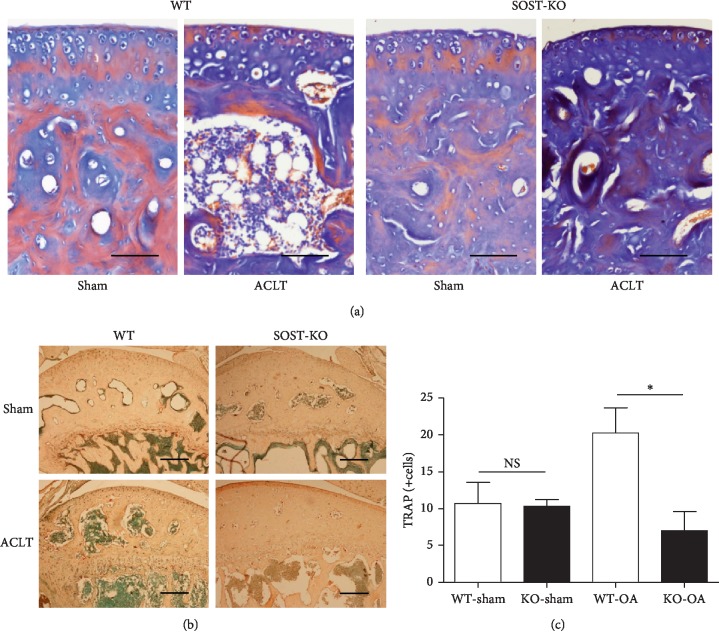
SOST KO mice presented an increase in activation of osteogenesis and a decrease in activation of osteoclasts. (a) The Masson trichrome staining revealed that the mineralization in the subchondral area was more intense in the OA knees of SOST KO mice, when compared to that of WT mice. (b, c) The TRAP-positive cells were identified as red multinuclear cells near the subchondral trabecular bone. The TRAP-positive cell number in OA knees of SOST KO mice was comparatively lesser than that of WT mice. There was no significant difference between the sham-operation groups of SOST KO and WT mice (^*∗*^*P* < 0.05; the data were presented as mean ± SD).

Masson staining and TRAP staining were used to assess the osteogenic and osteoclastic activities in mouse knees. As revealed by the Masson staining, ACLT induced the active osteogenesis in both the subchondral bone plate and trabecular bone area of WT and SOST KO mice (Figures 4(a) and 4(b)). Furthermore, SOST KO mice exhibited greater activation of osteogenesis in ACLT knees, when compared with their WT counterparts (Figure 4(a)). The number of TRAP-positive cells, which represents the osteoclasts, in SOST KO mice was significantly lower than those in WT mice (SOST KO *vs.* WT: 7.00 ± 2.65 *vs.* 20.25 ± 3.43, *P* < 0.05), indicating the decrease in osteoclast activity (Figures 4(b) and 4(c)).

## 4. Discussion

The present study demonstrated that the sclerostin expression pattern was the same in the articular cartilage and subchondral bone in response to the joint instability induced by ACLT, and SOST-KO mice developed a severer OA phenotype than WT mice shortly after surgery, with an increased osteogenic activity and decreased osteoclast activity in both the subchondral bone plate and subchondral trabecular bone as well as the catabolic activity in cartilage. These results indicate that SOST deficiency aggravates osteoarthritis in mice by promoting the sclerosis of the subchondral unit.

It is widely known that the expression of sclerostin by osteocytes is regulated by mechanical loading. Bouaziz et al. [13] reported a higher BMD and BV/TV ratio in the subchondral bone of SOST KO mice, when compared to WT mice, on the sham-operation side, and this arrived to the conclusion that sclerostin inhibits bone formation in the subchondral bone in the early stages of OA.

A previous study revealed that the genetic loss of sclerostin in mice has no effect on cartilage integrity in aging knee joints [20]. Nevertheless, recent studies have emerged with evidences that might be able to verify whether sclerostin plays a protective role in maintaining cartilage integrity in OA [12, 13, 21]. Chan et al. [12] reported that sclerostin generated an opposing effect on subchondral bone and cartilage. A recent study conducted by Chang et al. [14] had found sclerostin upregulated in articular cartilage postinjury from day 0 to day 3, treating SOST^TG^ and WT injured joints with the recombinant SOST protein reduced level of activated MMPs postinjury, to conclude that sclerostin functions as a protective agent after joint injury to prevent cartilage degradation. However, in the present study, the objective, the time frame, and conclusions are different, and the expression of sclerostin in each structure of the osteochondral unit was simultaneously presented. Sclerostin expression in the joint was found to be upregulated shorty after ACLT from week 0 to week 2 and subsequently downregulated from week 2 to week 4. The same changes were simultaneously found in the calcified cartilage, subchondral bone plate, and subchondral trabecular bone. In addition, in the cartilage of SOST KO mice, MMP-13, which is known to degrade the cartilage extracellular matrix, significantly increased after ACLT compared to WT mice, while Col II, which is known as an anabolic marker, significantly decreased after ACLT compared to WT mice. These data suggest that the reaction of sclerostin to the mechanical stress induced by joint instability is the same in the subchondral unit. Thus, it can be concluded that sclerostin plays a protective role in development of OA by increased catabolic activity in both subchondral bone and calcified cartilage.

It is known that in, the late stage of OA, calcified cartilage advances into noncalcified cartilage, creating a footprint of tidemarks, and resulting in subchondral bone sclerosis and a more fibrillated articular cartilage [22]. The inhomogeneity in the density and stiffness of the subchondral bone could exacerbate the shear stresses, thereby causing the cartilage to deform [23, 24]. sssssInterestingly, in this present study, it was found that the contour of the tide mark that separates the calcified cartilage from noncalcified cartilage was distinctly distorted in SOST KO mice, when compared to WT mice, indicating that suppressing sclerostin expression not only affects bone remodeling but also affects subchondral bone structure. Thus, SOST/sclerostin aggravated OA by disturbing the homogeneities of the subchondral bone and creating stress concentrating beneath the cartilage, thereby adversely affecting the overlying calcified and articular cartilage. Jia et al.'s [21] data can further support this indication. They had reported that the mechanical loading-induced attenuation of sclerostin expression and elevation of bone formation along the SBP surface are the major characterizing subchondral bone phenotypes associated with severe late-stage OA in mice. Furthermore, it was shown in the present study that, under the circumstances of surgery-induced OA, the balance of bone formation and bone resorption was interfered. Compared with OA knees in WT mice, SOST KO mice presented with a more activated status of osteogenesis, with an increased rate of bone formation and a decreased rate of bone resorption, indicating that the effect of sclerostin is probably correlated to the changes of the subchondral bone.

## 5. Conclusion

The present study presents the role of the SOST gene in OA subchondral bone sclerosis and its impact on the severity of OA. The following conclusions were drawn: the SOST gene and its encoded protein sclerostin occurs in both calcified cartilage and subchondral bone of the joint, and the deficiency of SOST/sclerostin aggravates the OA phenotype by increasing osteogenic activity in subchondral bone and increasing catabolic activity of cartilage, suggesting the protective role of SOST/sclerostin in the early stage of OA.

## Data Availability

The data used to support the findings of this study are included within the article.
